# Ethyl 5-{[(*E*)-2-(isonicotinoyl)hydrazinyl­idene]methyl}-3,4-dimethyl-1*H*-pyrrole-2-carboxyl­ate dihydrate

**DOI:** 10.1107/S1600536811017132

**Published:** 2011-05-14

**Authors:** Zhao-Po Zhang, Yuan Wang, Ming-Jia Lu, Lei-Wei Jia, Hong-Chang Qiao

**Affiliations:** a Department of Physics and Chemistry, Henan Polytechnic University, Jiaozuo 454000, People’s Republic of China

## Abstract

In the title compound, C_16_H_18_N_4_O_3_·2H_2_O, the dihedral angle between the pyrrole and pyridine rings in the hydrazone mol­ecule is 7.12 (3)°. In the crystal structure, inter­molecular N—H⋯O, O—H⋯N and O—H⋯O hydrogen bonds link the hydrazone and water mol­ecules into double layers parallel to (101). The crystal packing exhibits weak π–π inter­actions between the pyrrole and pyridine rings of neighbouring hydrazone mol­ecules [centroid–centroid distance = 3.777 (3) Å]. The crystal studied was a non-merohedral twin, the refined ratio of twin domains being 0.73 (3):0.27 (3).

## Related literature

For the anti­oxidant and DNA-binding properties of hydrazone complexes, see: Liu & Yang (2009 ▶). For the synthesis and structure of 5-formyl-3,4-dimethyl-1*H*-pyrrole-2-carboxyl­ate, see: Wu *et al.* (2009 ▶). For the similar structure of ethyl 5-[(3,4-dimethyl-1*H*-pyrrole-2-carboxyl­imino)-meth­yl]-3,4-dimethyl-1*H*-pyrrole-2-carboxyl­ate monohydrate, see: Wang *et al.* (2009 ▶).
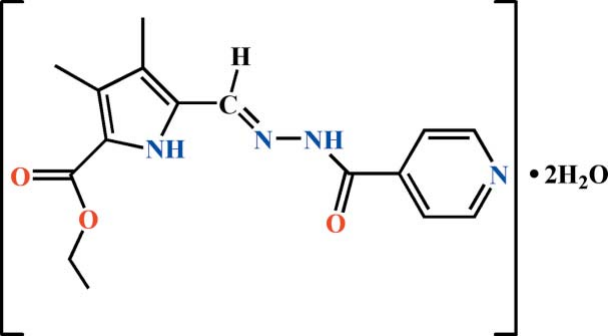


## Experimental

### 

#### Crystal data

C_16_H_18_N_4_O_3_·2H_2_O
*M* *_r_* = 350.38
Monoclinic, 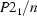

*a* = 8.297 (4) Å
*b* = 18.120 (6) Å
*c* = 11.834 (4) Å
β = 91.814 (4)°
*V* = 1778.3 (11) Å^3^
*Z* = 4
Mo *K*α radiation
μ = 0.10 mm^−1^
*T* = 296 K
0.23 × 0.21 × 0.16 mm

#### Data collection

Bruker SMART APEX CCD diffractometer
Absorption correction: multi-scan (*SADABS*; Bruker, 2007 ▶) *T*_min_ = 0.977, *T*_max_ = 0.984
10998 measured reflections
3839 independent reflections
2671 reflections with *I* > 2σ(*I*)
*R*_int_ = 0.037

#### Refinement

*R*[*F* ^2^ > 2σ(*F* ^2^)] = 0.074
*wR*(*F* ^2^) = 0.176
*S* = 1.02
3839 reflections
241 parameters
H atoms treated by a mixture of independent and constrained refinement
Δρ_max_ = 0.41 e Å^−3^
Δρ_min_ = −0.27 e Å^−3^

### 

Data collection: *APEX2* (Bruker, 2007 ▶); cell refinement: *SAINT* (Bruker, 2007 ▶); data reduction: *SAINT*; program(s) used to solve structure: *SHELXS97* (Sheldrick, 2008 ▶); program(s) used to refine structure: *SHELXL97* (Sheldrick, 2008 ▶); molecular graphics: *SHELXTL* (Sheldrick, 2008 ▶); software used to prepare material for publication: *SHELXTL*.

## Supplementary Material

Crystal structure: contains datablocks I, global. DOI: 10.1107/S1600536811017132/cv5084sup1.cif

Structure factors: contains datablocks I. DOI: 10.1107/S1600536811017132/cv5084Isup2.hkl

Additional supplementary materials:  crystallographic information; 3D view; checkCIF report

## Figures and Tables

**Table 1:** Hydrogen-bond geometry (Å, °)

*D*—H⋯*A*	*D*—H	H⋯*A*	*D*⋯*A*	*D*—H⋯*A*
N2—H2*A*⋯O1*W*^i^	0.86	2.08	2.919 (4)	164
N4—H4*A*⋯O2*W*	0.86	2.24	3.084 (4)	165
O1*W*—H1*C*⋯N1	0.69 (5)	2.18 (5)	2.854 (4)	164 (6)
O1*W*—H1*D*⋯O2*W*^ii^	0.91 (5)	1.88 (5)	2.790 (5)	174 (5)
O2*W*—H2*C*⋯O2^iii^	0.92 (5)	2.06 (5)	2.941 (4)	160 (4)
O2*W*—H2*D*⋯O1	0.82 (5)	2.27 (5)	3.048 (4)	159 (5)
O2*W*—H2*D*⋯N3	0.82 (5)	2.43 (5)	3.014 (4)	129 (4)

Symmetry codes: (i) 
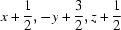
; (ii) 
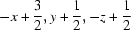
; (iii) 
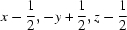
.

**Fig. 1.**
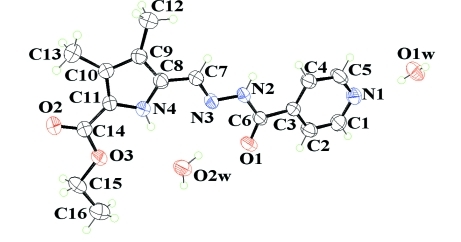
The title compound with the displacement ellipsoids shown at the 50% probability level.

## supplementary crystallographic information

### Comment

In recent years, hydrazone complexes have received extensive attention due to their biological and pharmaceutical activities (Liu *et al.*, 2009). As a part of our studies of hydrazone ligands bearing pyrrole unit (Wang *et al.*, 2009), we present here the crystal structure of the title compound.

In the title compound (Fig. 1), the imine C=N double bond has an E configuration. The dihedral angle between pyrrole (N4/C8–C11, r.m.s. deviation 0.0030 Å) and pyridine rings (N1/C1–C5, r.m.s. deviation 0.0038 Å) in the hydrazone molecule is 7.12 (3)°. In the crystal structure, intermolecular N—H···O, O—H···N and O—H···O hydrogen bonds link the hydrazone and water molecules into doubled layers parallel to (101) plane. The crystal packing exhibits weak π–π interactions between the pyrrole and pyridine rings from the neighbouring hydrazone molecules [centroid-to-centroid distance of 3.777 (3) Å].

### Experimental

Isonicotinohydrazide (0.137 g, 1 mmol) and ethyl 5-formyl-3,4-dimethyl-1*H*-pyrrole-2-carboxylate (0.167 g, 1 mmol) (Wu *et al.*, 2009) was dissolved in an ethanol solution. The mixture was stirred for 4 h at room temperture. The resulting solution was left in air for a few days, yielding yellow prism-shaped crystals.

### Refinement

The water H atoms were located in a difference Fourier map and refined with *U*_iso_(H)=1.5Ueq(O). Other H atoms were placed in calculated positions, with C—H = 0.93–0.97 Å and N—H = 0.86 Å, and were thereafter treated as riding, with *U*_iso_(H) values of 1.5Ueq(C) for methyl groups and 1.2Ueq(C,*N*) for others. The crystal studied was a twin.

### Crystal data

**Table:**

C_16_H_18_N_4_O_3_·2H_2_O	*F*(000) = 744
*M**_r_* = 350.38	*D*_x_ = 1.309 Mg m^−^^3^
Monoclinic, *P*2_1_/*n*	Mo *K*α radiation, λ = 0.71073 Å
*a* = 8.297 (4) Å	θ = 2.7–25.3°
*b* = 18.120 (6) Å	µ = 0.10 mm^−^^1^
*c* = 11.834 (4) Å	*T* = 296 K
β = 91.814 (4)°	Prism, yellow
*V* = 1778.3 (11) Å^3^	0.23 × 0.21 × 0.16 mm
*Z* = 4	

### Data collection

**Table:**

Bruker SMART APEX CCD diffractometer	3839 independent reflections
Radiation source: fine-focus sealed tube	2671 reflections with *I* > 2σ(*I*)
graphite	*R*_int_ = 0.037
φ and ω scans	θ_max_ = 27.0°, θ_min_ = 2.1°
Absorption correction: multi-scan (*SADABS*; Bruker, 2007)	*h* = −10→10
*T*_min_ = 0.977, *T*_max_ = 0.984	*k* = −9→24
10998 measured reflections	*l* = −15→15

### Refinement

**Table:**

Refinement on *F*^2^	Primary atom site location: structure-invariant direct methods
Least-squares matrix: full	Secondary atom site location: difference Fourier map
*R*[*F*^2^ > 2σ(*F*^2^)] = 0.074	Hydrogen site location: inferred from neighbouring sites
*wR*(*F*^2^) = 0.176	H atoms treated by a mixture of independent and constrained refinement
*S* = 1.02	*w* = 1/[σ^2^(*F*_o_^2^) + (0.02*P*)^2^ + 3.5*P*] where *P* = (*F*_o_^2^ + 2*F*_c_^2^)/3
3839 reflections	(Δ/σ)_max_ = 0.069
241 parameters	Δρ_max_ = 0.41 e Å^−^^3^
0 restraints	Δρ_min_ = −0.27 e Å^−^^3^

### Special details

**Table:**

Geometry. All e.s.d.'s (except the e.s.d. in the dihedral angle between two l.s. planes) are estimated using the full covariance matrix. The cell e.s.d.'s are taken into account individually in the estimation of e.s.d.'s in distances, angles and torsion angles; correlations between e.s.d.'s in cell parameters are only used when they are defined by crystal symmetry. An approximate (isotropic) treatment of cell e.s.d.'s is used for estimating e.s.d.'s involving l.s. planes.
Refinement. Refinement of *F*^2^ against ALL reflections. The weighted *R*-factor *wR* and goodness of fit *S* are based on *F*^2^, conventional *R*-factors *R* are based on *F*, with *F* set to zero for negative *F*^2^. The threshold expression of *F*^2^ > σ(*F*^2^) is used only for calculating *R*-factors(gt) *etc*. and is not relevant to the choice of reflections for refinement. *R*-factors based on *F*^2^ are statistically about twice as large as those based on *F*, and *R*- factors based on ALL data will be even larger.

### Fractional atomic coordinates and isotropic or equivalent isotropic displacement parameters (Å^2^)

**Table:**

	*x*	*y*	*z*	*U*_iso_*/*U*_eq_	
O1	0.6319 (3)	0.46189 (12)	0.4160 (2)	0.0530 (6)	
O2	1.1091 (4)	0.29040 (14)	0.9756 (2)	0.0660 (8)	
O3	0.9696 (3)	0.27423 (12)	0.8122 (2)	0.0564 (7)	
N1	0.4775 (4)	0.68078 (16)	0.1780 (2)	0.0532 (8)	
N2	0.7766 (3)	0.55247 (14)	0.5019 (2)	0.0406 (6)	
H2A	0.8043	0.5982	0.5024	0.049*	
N3	0.8374 (3)	0.50452 (14)	0.5828 (2)	0.0420 (6)	
N4	0.9761 (3)	0.41573 (14)	0.7584 (2)	0.0415 (6)	
H4A	0.9158	0.3892	0.7141	0.050*	
C1	0.4299 (4)	0.6106 (2)	0.1836 (3)	0.0545 (9)	
H1	0.3507	0.5946	0.1319	0.065*	
C2	0.4901 (4)	0.56074 (19)	0.2606 (3)	0.0498 (8)	
H2	0.4522	0.5124	0.2604	0.060*	
C3	0.6076 (4)	0.58255 (17)	0.3388 (3)	0.0385 (7)	
C4	0.6579 (5)	0.65506 (18)	0.3348 (3)	0.0531 (9)	
H4	0.7355	0.6730	0.3861	0.064*	
C5	0.5898 (5)	0.70012 (19)	0.2527 (3)	0.0605 (10)	
H5	0.6267	0.7485	0.2500	0.073*	
C6	0.6727 (4)	0.52615 (16)	0.4219 (3)	0.0380 (7)	
C7	0.9388 (4)	0.53192 (17)	0.6540 (3)	0.0418 (7)	
H7A	0.9686	0.5811	0.6471	0.050*	
C8	1.0084 (4)	0.48875 (17)	0.7448 (3)	0.0412 (7)	
C9	1.1112 (4)	0.51125 (18)	0.8322 (3)	0.0428 (8)	
C10	1.1406 (4)	0.44984 (18)	0.9021 (3)	0.0423 (7)	
C11	1.0555 (4)	0.39167 (17)	0.8543 (3)	0.0411 (7)	
C12	1.1778 (5)	0.5870 (2)	0.8501 (3)	0.0654 (11)	
H12A	1.1754	0.6131	0.7795	0.098*	
H12B	1.2870	0.5837	0.8789	0.098*	
H12C	1.1137	0.6129	0.9033	0.098*	
C13	1.2480 (5)	0.4481 (2)	1.0061 (3)	0.0589 (10)	
H13A	1.1894	0.4654	1.0695	0.088*	
H13B	1.3398	0.4792	0.9954	0.088*	
H13C	1.2835	0.3984	1.0201	0.088*	
C14	1.0502 (4)	0.31492 (18)	0.8893 (3)	0.0452 (8)	
C15	0.9548 (5)	0.19650 (18)	0.8342 (3)	0.0609 (10)	
H15A	0.8978	0.1883	0.9034	0.073*	
H15B	1.0605	0.1738	0.8420	0.073*	
C16	0.8642 (7)	0.1652 (2)	0.7371 (4)	0.0830 (15)	
H16A	0.7623	0.1900	0.7281	0.125*	
H16B	0.8461	0.1135	0.7498	0.125*	
H16C	0.9247	0.1715	0.6700	0.125*	
O1W	0.4291 (4)	0.80318 (15)	0.0290 (3)	0.0654 (8)	
H1C	0.428 (7)	0.770 (3)	0.059 (5)	0.098*	
H1D	0.528 (6)	0.813 (3)	0.001 (4)	0.098*	
O2W	0.7783 (3)	0.34071 (15)	0.5656 (2)	0.0588 (7)	
H2C	0.704 (6)	0.305 (3)	0.544 (4)	0.088*	
H2D	0.744 (6)	0.380 (3)	0.541 (4)	0.088*	

### Atomic displacement parameters (Å^2^)

**Table:**

	*U*^11^	*U*^22^	*U*^33^	*U*^12^	*U*^13^	*U*^23^
O1	0.0688 (16)	0.0297 (12)	0.0598 (15)	−0.0029 (11)	−0.0066 (12)	0.0035 (10)
O2	0.102 (2)	0.0431 (14)	0.0513 (15)	0.0040 (14)	−0.0203 (15)	0.0099 (12)
O3	0.0790 (18)	0.0327 (12)	0.0562 (15)	−0.0080 (12)	−0.0160 (13)	0.0066 (11)
N1	0.0622 (19)	0.0453 (17)	0.0512 (18)	0.0079 (14)	−0.0106 (15)	0.0081 (14)
N2	0.0489 (15)	0.0314 (13)	0.0412 (15)	0.0009 (11)	−0.0045 (12)	0.0071 (11)
N3	0.0514 (16)	0.0334 (14)	0.0412 (15)	0.0061 (12)	0.0007 (12)	0.0060 (11)
N4	0.0479 (15)	0.0373 (14)	0.0388 (14)	−0.0002 (12)	−0.0063 (12)	0.0009 (11)
C1	0.055 (2)	0.056 (2)	0.051 (2)	−0.0028 (17)	−0.0150 (17)	0.0018 (17)
C2	0.053 (2)	0.0374 (18)	0.058 (2)	−0.0064 (15)	−0.0077 (17)	0.0021 (16)
C3	0.0430 (17)	0.0327 (16)	0.0396 (17)	0.0022 (13)	0.0000 (13)	−0.0006 (13)
C4	0.069 (2)	0.0373 (18)	0.052 (2)	−0.0048 (16)	−0.0192 (18)	0.0029 (15)
C5	0.079 (3)	0.0298 (18)	0.072 (3)	−0.0034 (17)	−0.017 (2)	0.0099 (17)
C6	0.0437 (17)	0.0295 (16)	0.0410 (17)	0.0023 (13)	0.0040 (14)	0.0027 (13)
C7	0.0525 (19)	0.0333 (16)	0.0396 (17)	0.0013 (14)	0.0019 (15)	0.0003 (13)
C8	0.0459 (18)	0.0379 (17)	0.0399 (17)	0.0056 (14)	0.0024 (14)	0.0016 (14)
C9	0.0515 (19)	0.0367 (17)	0.0404 (17)	0.0011 (14)	0.0029 (15)	−0.0014 (13)
C10	0.0501 (19)	0.0384 (17)	0.0383 (17)	0.0027 (14)	−0.0022 (14)	−0.0018 (14)
C11	0.0502 (18)	0.0368 (17)	0.0359 (16)	0.0040 (14)	−0.0030 (14)	0.0018 (13)
C12	0.092 (3)	0.042 (2)	0.061 (2)	−0.008 (2)	−0.011 (2)	0.0002 (18)
C13	0.072 (3)	0.056 (2)	0.047 (2)	0.0001 (19)	−0.0138 (18)	−0.0002 (17)
C14	0.057 (2)	0.0384 (18)	0.0404 (18)	0.0043 (15)	−0.0020 (15)	0.0007 (14)
C15	0.086 (3)	0.0304 (18)	0.065 (2)	−0.0057 (18)	−0.004 (2)	0.0070 (16)
C16	0.118 (4)	0.043 (2)	0.086 (3)	−0.005 (2)	−0.025 (3)	−0.005 (2)
O1W	0.0706 (18)	0.0443 (16)	0.081 (2)	0.0111 (14)	−0.0049 (16)	0.0186 (14)
O2W	0.0681 (18)	0.0384 (14)	0.0692 (18)	0.0038 (12)	−0.0093 (14)	−0.0031 (13)

### Geometric parameters (Å, °)

**Table:**

O1—C6	1.214 (4)	C7—H7A	0.9300
O2—C14	1.204 (4)	C8—C9	1.382 (5)
O3—C14	1.336 (4)	C9—C10	1.403 (4)
O3—C15	1.438 (4)	C9—C12	1.492 (5)
N1—C5	1.312 (5)	C10—C11	1.380 (4)
N1—C1	1.334 (5)	C10—C13	1.497 (5)
N2—C6	1.347 (4)	C11—C14	1.452 (4)
N2—N3	1.377 (3)	C12—H12A	0.9600
N2—H2A	0.8600	C12—H12B	0.9600
N3—C7	1.273 (4)	C12—H12C	0.9600
N4—C8	1.361 (4)	C13—H13A	0.9600
N4—C11	1.366 (4)	C13—H13B	0.9600
N4—H4A	0.8600	C13—H13C	0.9600
C1—C2	1.366 (5)	C15—C16	1.468 (5)
C1—H1	0.9300	C15—H15A	0.9700
C2—C3	1.380 (4)	C15—H15B	0.9700
C2—H2	0.9300	C16—H16A	0.9600
C3—C4	1.380 (4)	C16—H16B	0.9600
C3—C6	1.506 (4)	C16—H16C	0.9600
C4—C5	1.376 (5)	O1W—H1C	0.69 (5)
C4—H4	0.9300	O1W—H1D	0.91 (5)
C5—H5	0.9300	O2W—H2C	0.92 (5)
C7—C8	1.435 (4)	O2W—H2D	0.82 (5)
			
C14—O3—C15	117.4 (3)	C11—C10—C9	106.7 (3)
C5—N1—C1	115.2 (3)	C11—C10—C13	127.2 (3)
C6—N2—N3	118.5 (3)	C9—C10—C13	126.1 (3)
C6—N2—H2A	120.7	N4—C11—C10	108.9 (3)
N3—N2—H2A	120.7	N4—C11—C14	121.6 (3)
C7—N3—N2	115.7 (3)	C10—C11—C14	129.4 (3)
C8—N4—C11	108.5 (3)	C9—C12—H12A	109.5
C8—N4—H4A	125.8	C9—C12—H12B	109.5
C11—N4—H4A	125.8	H12A—C12—H12B	109.5
N1—C1—C2	124.1 (3)	C9—C12—H12C	109.5
N1—C1—H1	117.9	H12A—C12—H12C	109.5
C2—C1—H1	117.9	H12B—C12—H12C	109.5
C1—C2—C3	119.6 (3)	C10—C13—H13A	109.5
C1—C2—H2	120.2	C10—C13—H13B	109.5
C3—C2—H2	120.2	H13A—C13—H13B	109.5
C4—C3—C2	117.2 (3)	C10—C13—H13C	109.5
C4—C3—C6	124.5 (3)	H13A—C13—H13C	109.5
C2—C3—C6	118.3 (3)	H13B—C13—H13C	109.5
C3—C4—C5	118.1 (3)	O2—C14—O3	123.9 (3)
C3—C4—H4	120.9	O2—C14—C11	125.5 (3)
C5—C4—H4	120.9	O3—C14—C11	110.7 (3)
N1—C5—C4	125.7 (3)	O3—C15—C16	106.3 (3)
N1—C5—H5	117.1	O3—C15—H15A	110.5
C4—C5—H5	117.1	C16—C15—H15A	110.5
O1—C6—N2	123.4 (3)	O3—C15—H15B	110.5
O1—C6—C3	121.3 (3)	C16—C15—H15B	110.5
N2—C6—C3	115.3 (3)	H15A—C15—H15B	108.7
N3—C7—C8	121.7 (3)	C15—C16—H16A	109.5
N3—C7—H7A	119.2	C15—C16—H16B	109.5
C8—C7—H7A	119.2	H16A—C16—H16B	109.5
N4—C8—C9	108.5 (3)	C15—C16—H16C	109.5
N4—C8—C7	122.9 (3)	H16A—C16—H16C	109.5
C9—C8—C7	128.6 (3)	H16B—C16—H16C	109.5
C8—C9—C10	107.4 (3)	H1C—O1W—H1D	112 (5)
C8—C9—C12	126.5 (3)	H2C—O2W—H2D	107 (4)
C10—C9—C12	126.1 (3)		
			
C6—N2—N3—C7	177.9 (3)	C7—C8—C9—C10	177.6 (3)
C5—N1—C1—C2	0.3 (6)	N4—C8—C9—C12	179.8 (3)
N1—C1—C2—C3	0.1 (6)	C7—C8—C9—C12	−1.8 (6)
C1—C2—C3—C4	0.3 (5)	C8—C9—C10—C11	0.4 (4)
C1—C2—C3—C6	−179.4 (3)	C12—C9—C10—C11	179.9 (3)
C2—C3—C4—C5	−1.0 (5)	C8—C9—C10—C13	178.8 (3)
C6—C3—C4—C5	178.7 (3)	C12—C9—C10—C13	−1.8 (6)
C1—N1—C5—C4	−1.1 (6)	C8—N4—C11—C10	−0.5 (4)
C3—C4—C5—N1	1.5 (7)	C8—N4—C11—C14	−177.4 (3)
N3—N2—C6—O1	−1.4 (5)	C9—C10—C11—N4	0.0 (4)
N3—N2—C6—C3	178.1 (3)	C13—C10—C11—N4	−178.3 (3)
C4—C3—C6—O1	−174.7 (3)	C9—C10—C11—C14	176.6 (3)
C2—C3—C6—O1	5.0 (5)	C13—C10—C11—C14	−1.7 (6)
C4—C3—C6—N2	5.8 (5)	C15—O3—C14—O2	−0.7 (5)
C2—C3—C6—N2	−174.5 (3)	C15—O3—C14—C11	179.5 (3)
N2—N3—C7—C8	178.2 (3)	N4—C11—C14—O2	−175.6 (3)
C11—N4—C8—C9	0.8 (4)	C10—C11—C14—O2	8.2 (6)
C11—N4—C8—C7	−177.7 (3)	N4—C11—C14—O3	4.3 (5)
N3—C7—C8—N4	2.6 (5)	C10—C11—C14—O3	−172.0 (3)
N3—C7—C8—C9	−175.5 (3)	C14—O3—C15—C16	−179.3 (4)
N4—C8—C9—C10	−0.8 (4)		

### Hydrogen-bond geometry (Å, °)

**Table:**

*D*—H···*A*	*D*—H	H···*A*	*D*···*A*	*D*—H···*A*
N2—H2A···O1W^i^	0.86	2.08	2.919 (4)	164
N4—H4A···O2W	0.86	2.24	3.084 (4)	165
O1W—H1C···N1	0.69 (5)	2.18 (5)	2.854 (4)	164 (6)
O1W—H1D···O2W^ii^	0.91 (5)	1.88 (5)	2.790 (5)	174 (5)
O2W—H2C···O2^iii^	0.92 (5)	2.06 (5)	2.941 (4)	160 (4)
O2W—H2D···O1	0.82 (5)	2.27 (5)	3.048 (4)	159 (5)
O2W—H2D···N3	0.82 (5)	2.43 (5)	3.014 (4)	129 (4)

Symmetry codes: (i) *x*+1/2, −*y*+3/2, *z*+1/2; (ii) −*x*+3/2, *y*+1/2, −*z*+1/2; (iii) *x*−1/2, −*y*+1/2, *z*−1/2.
